# From prediction to practice: mitigating bias and data shift in machine-learning models for chemotherapy-induced organ dysfunction across unseen cancers

**DOI:** 10.1136/bmjonc-2024-000430

**Published:** 2024-11-02

**Authors:** Matthew Watson, Pinkie Chambers, Luke Steventon, James Harmsworth King, Angelo Ercia, Heather Shaw, Noura Al Moubayed

**Affiliations:** 1 Department of Computer Science, Durham University, Durham, UK; 2 Cancer Division, University College London Hospitals NHS Foundation Trust, London, UK; 3 School of Pharmacy, University College London, London, UK; 4 Evergreen Life Ltd, Manchester, UK; 5 Mount Vernon Cancer Centre, Northwood, UK

**Keywords:** Adverse effects, Chemotherapy

## Abstract

**Objectives:**

Routine monitoring of renal and hepatic function during chemotherapy ensures that treatment-related organ damage has not occurred and clearance of subsequent treatment is not hindered; however, frequency and timing are not optimal. Model bias and data heterogeneity concerns have hampered the ability of machine learning (ML) to be deployed into clinical practice. This study aims to develop models that could support individualised decisions on the timing of renal and hepatic monitoring while exploring the effect of data shift on model performance.

**Methods and analysis:**

We used retrospective data from three UK hospitals to develop and validate ML models predicting unacceptable rises in creatinine/bilirubin post cycle 3 for patients undergoing treatment for the following cancers: breast, colorectal, lung, ovarian and diffuse large B-cell lymphoma.

**Results:**

We extracted 3614 patients with no missing blood test data across cycles 1–6 of chemotherapy treatment. We improved on previous work by including predictions post cycle 3. Optimised for sensitivity, we achieve F2 scores of 0.7773 (bilirubin) and 0.6893 (creatinine) on unseen data. Performance is consistent on tumour types unseen during training (F2 bilirubin: 0.7423, F2 creatinine: 0.6820).

**Conclusion:**

Our technique highlights the effectiveness of ML in clinical settings, demonstrating the potential to improve the delivery of care. Notably, our ML models can generalise to unseen tumour types. We propose gold-standard bias mitigation steps for ML models: evaluation on multisite data, thorough patient population analysis, and both formalised bias measures and model performance comparisons on patient subgroups. We demonstrate that data aggregation techniques have unintended consequences on model bias.

**WHAT IS ALREADY KNOWN ON THIS TOPIC**

- Previous work has shown, as a proof of concept, that machine learning (ML) can be used to predict renal and hepatic dysfunction in chemotherapy patients at the third cycle of treatment. However, data were limited to a small number of patients and tumour types, and for a single cycle of treatment.

**WHAT THIS STUDY ADDS**

- We propose improved modelling techniques that predict dysfunction at multiple cycles (cycles 3–6 inclusive) and, through the inclusion of data from five cancer types, across three cancer departments in different hospitals, extensively analyse the generalisability and perceived bias of our technique. Our findings demonstrate the generalisability of our technique to cancer diagnoses and patient populations not included in the training data.

**HOW THIS STUDY MIGHT AFFECT RESEARCH, PRACTICE OR POLICY**

- Our proposed chemotherapy risk stratification models improve the appropriateness of cancer care, meaning that patients would have more personalised blood monitoring before chemotherapy. This could significantly reduce pressure, particularly in areas with limited access to phlebotomy services. The generalisability of our technique to unseen cancer diagnoses mitigates the requirement for extensive and expensive training on data that encompasses all types of tumours. Our work also shows that data shift and bias analysis methods should become standard practice in healthcare ML research due to the prevalence of these issues in medical data.

## Background

 Machine learning (ML) has seen rapid growth over the past decade,^1^ including within the field of healthcare.^2^ In the research setting, ML techniques have been successfully applied to a wide range of medical tasks, such as chest X-ray diagnosis,^3^ electronic health record analysis^4^ and computational biology,^5^ with some approaches beginning to outperform medical experts.^6^ However, this is yet to be translated to medical practice; although the US Food and Drugs Administration has thus far approved 343 ML-based medical devices,^7^ this is eclipsed by the number of research papers published in the field that claim positive results.^8^

Alongside advancements in applying ML to healthcare tasks, research working with clinicians, patients and policy-makers has attempted to codify necessary criteria for clinical deployment.^9^ Models must be transparent and interpretable,^10 11^ identify and mitigate bias^12^ and be robust and secure,^13^ all while maintaining high performance. These are the main barriers that ML in healthcare faces; while all studies report their techniques’ performance in terms of model accuracy, it is rare for these complementary criteria to be analysed, hindering their use in practice.

Indeed, many ML techniques suffer from problems that directly affect these areas. For example, it has been shown that deep-learning approaches are highly susceptible to minor input changes^14^ and hyperparameter variations,^15 16^ raising concerns about model robustness. There is also an increasing awareness of neural networks making biased decisions,^17^ such as black patients being assigned the same risk level as white patients, despite being sicker than their white counterparts.^18^ While the underlying source of this bias can vary,^19^ a common culprit is the gap between the training data distribution and that of the real data to which it will be applied,^20^ along with the model’s ability to generalise well^21^ to this unseen data.

This mismatch (ie, data shift) between training and real-world data is especially prevalent in healthcare.^22^ The cost, time and privacy constraints associated with medical data limit access to diverse datasets, often leading to ML models trained on data from only a single hospital.^23^ This can lead to the model making biased decisions and limit generalisability, for example, due to varying patient populations (eg, the level of poverty in a hospital’s catchment area can greatly affect the general health of its patients^24^) or standard operating procedures that affect both patient outcomes and the type and quality of data collected. Furthermore, in terms of population health, behaviours, treatments and outcomes will gradually change over time, possibly resulting in the model’s accuracy decreasing should it not be continually updated.^25^

Limited diversity in publicly available healthcare data hinders bias identification and mitigation,^26^ and these data and research gaps often impede regulatory approval for clinical use.^27 28^ Although some techniques have been proposed to address bias^29 30^ these are very much still in their infancy. This paper explores these issues through the lens of chemotherapy patient risk stratification, providing concrete examples of when and why these issues occur, how to detect them and guidance for addressing them.

We achieve this by significantly building on existing work on ML-based chemotherapy risk stratification,^31^ with the aim of detecting patients at risk of experiencing renal and/or hepatic dysfunction on commencement of treatment. These drug-related adverse effects occur in around 10% of patients^31^; the current model of care is to routinely undertake blood monitoring for all patients during a course of chemotherapy. This monitoring can result in lengthy delays for patients on the days they are receiving treatment.^32^ An ML model that is able to accurately stratify patients according to their risk of experiencing renal or hepatic deterioration during treatment would allow only patients at high risk of adverse effects to undergo blood tests prior to every treatment, resulting in benefits for both the patient experience and the cost of delivering care.^31 32^

ML has been used in chemotherapy for tasks such as dose delay^32^ and mortality prediction,^33^ however, the estimation of renal and hepatic dysfunction has gone relatively unexplored. Previous studies^31^ were limited to a small number of patients, focused on a single treatment cycle and three types of cancer. Our aim was to improve on these methods by introducing ML models that can accurately predict renal and hepatic deterioration at cycles 3–6, and to extensively evaluate our techniques on retrospective data from three large cancer departments in hospitals in the UK, each with distinct patient populations. By using datasets from three distinct hospitals (n=3614), we created models that are more generalisable than previous techniques. Additionally, we explore data shifts in the models to understand implications for future use.

## Methods

We retrospectively collected patient data from cancer departments within three UK hospitals (hospitals 1–3), each situated in a different area of the UK; all data were anonymised and underwent local governance review and data sharing agreements. Patients were extracted from the electronic prescribing (EP) system according to the inclusion criteria: patients were included if they were aged 18 or over and were receiving first-line chemotherapy treatment for colorectal cancer, diffuse large B-cell lymphoma (DLBCL) or early-stage (stages 1–3) breast cancer. Data were extracted for the period 1 January 2013–31 December 2018. Patients were followed for the first six treatment cycles (though the sixth cycle is not necessarily their last) and were excluded if they only received one cycle of treatment or the second cycle was more than 60 days after the first (online supplemental figure 1). These data extraction yielded 999, 530 and 1840 records from hospitals 1, 2 and 3, respectively. Patients with missing blood test data were then excluded (although missing demographic data, eg, comorbidities, were allowed); across all hospitals in the study, the rate of missing blood test data increased with treatment progression, especially in hospital 2 (online supplemental figure 2). After removing records with missing data, we were left with 627 (hospital 1A), 144 (hospital 2) and 1280 (hospital 3) patients. To assess the impact of temporal data shift on our proposed techniques, as well as their performance on cancer types not included in the training data, we subsequently extracted an additional 1563 records from hospital 1 consisting of patients receiving chemotherapy for breast cancer, bowel cancer, lung cancer or ovarian cancer. These data were extracted for the period 3 February 2020–8 November 2023. To distinguish between the two datasets from hospital 1, we name the original data extraction hospital 1A and the second hospital 1B.

We extracted patient demographics, blood test results and treatment details from the EP system. Blood test results at each cycle of treatment, as well as a baseline collected prior to the treatment starting, are used that contain: creatinine, bilirubin, haemoglobin, absolute neutrophil count and alanine aminotransferase levels. These tests were selected for their routine inclusion in clinical practice and established roles in toxicity prediction.^33 34^ Additional demographic data included age, sex, ethnicity, height, weight, cancer type, treatment regimen and relative dose intensity. The following comorbidities were extracted—diabetes, cardiovascular, thyroid, respiratory, arthritis and autoimmune diseases—however, the degree to which these data are collected varied significantly across hospitals. We conducted a comprehensive analysis of data shift, with summary statistics for each dataset presented in table 1. Online supplemental section 2 further explores these data distributions and discusses potential explanations for hospital-specific variation as well as the importance of addressing data shift when training and validating healthcare ML models.

**Table 1:** Summary statistics for each of the datasets used throughout the study

Parameter	Hospital 1A	Hospital 2	Hospital 3	Hospital 1B
Number of patients	627	144	1280	1563
Age	Median: 55 Range: 18–88	Median: 62 Range: 18–84	Median: 60 Range: 18–86	Median: 64 Range: 18–90
Sex	Female: 440 (70.2%) Male: 187 (29.8%)	Female: 78 (54.2%) Male: 66 (45.8%)	Female: 725 (59.0%) Male: 525 (41.0%)	Female: 1308 (83.7%) Male: 255 (16.3%)
Tumour type	Breast: 249 (39.7%) Bowel: 216 (34.4%) DLBCL: 162 (25.8%) Lung: 0 (0%) Ovarian: 0 (0%)	Breast: 19 (13.1%) Bowel: 98 (68.1%) DLBCL: 27 (18.8%) Lung: 0 (0%) Ovarian: 0 (0%)	Breast: 426 (33.3%) Bowel: 744 (58.1%) DLBCL: 110 (8.6%) Lung: 0 (0%) Ovarian: 0 (0%)	Breast: 470 (30.1%) Bowel: 358 (22.9%) DLBCL: 0 (0%) Lung: 126 (8.1%) Ovarian: 609 (38.9%)
Creatinine grade changes	36 (4%)	17 (12%)	215 (17%)	104 (7%)
Bilirubin grade changes	59 (11%)	24 (17%)	184 (14%)	92 (6%)

Patients with any missing blood test data in cycles 1–6 have been removed. Creatinine/bilirubin grade changes are only recorded if they are towards toxicity; if a patient experiences multiple grade changes (ie, at different cycles), they are only recorded once (at the cycle of their first change).

DLBCL, diffuse large B-cell lymphoma.

Improving on previous studies,^31 35^ we trained models to predict renal and/or hepatic dysfunction during cycles 3–6 inclusive, exploring two approaches:

1. Predict dysfunction at cycle *n* using demographics and lab results from cycles 1 and 2.
2. Predict dysfunction at cycle *n* using demographics and lab results from cycles 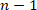 and 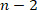.

We initially compared both and ultimately chose the best-performing technique to study in-depth in Results and Model Bias Analysis. The prediction of grade changes at cycle 3 onwards was chosen as many toxicities occur during the first cycle of treatment, and we believe that it would not be clinically acceptable to remove blood test monitoring at cycle 2.

### Model training and evaluation

Instead of a single model, we framed the problem as two separate regression tasks, predicting blood creatinine and bilirubin levels; each model’s output then guides patient stratification into high-risk and low-risk groups. This allows fine-grained control over risk levels as the decision threshold (and thus sensitivity) of our technique can be easily adjusted—prioritising avoiding false negatives (FNs), which could lead to poorer outcomes than false positives (FP). This threshold is based on the Common Terminology Criteria for Adverse Events^36^ (CTCAE), which defines abnormal grade changes for both creatinine and bilirubin; Results reports the resulting thresholds used after optimising for a lower FN rate. Online supplemental figure 3 presents a typical patient’s flow through the system.

We train Gradient Boosted Decision Trees (GBDTs) using the XGBoost library^37^ due to their state-of-the-art performance on tabular data^38 39^ and enhanced interpretability^40^ when compared with deep learning models. Furthermore, we chose GBDTs as they show increased performance when compared with multilayer perceptrons on our task (online supplemental section 2). For both creatinine and bilirubin prediction, we trained three separate GBDTs: (1) trained on hospital 1A data only, (2) trained on hospital 3 data only and (3) trained on combined data from hospitals 1A and 3. Comparing each of these models allowed us to investigate the impact of training data distribution on model performance.

Data from hospitals 1A and 3 were split 80/20 for training/testing, while hospital 1B and hospital 2 data were used as an unseen validation set to assess temporal and intersite generalisability, respectively. Hospital 1B data were further stratified by cancer diagnosis; the first set comprised patients with breast cancer, bowel cancer or DLBCL, while the second set included patients with lung or ovarian cancers. As lung and ovarian tumours were unrepresented in the training data, this specifically evaluated the model’s ability to generalise to unseen tumour types. All data were standardised by removing the mean and scaling to unit variance prior to being used and 10-fold cross-validation was used for training, along with a Tree Parzen Estimator-based hyperparameter search. The hyperparameter search space is shown in online supplemental table 4, and the final model hyperparameters are in online supplemental table 5. Due to the small number of features available in our dataset, no feature selection techniques were used.

We initially focused on predicting cycle 3 grade changes only for direct comparison with previous work,^31 35^ later extending models to cycles 4–6. We primarily used the F2 score as a measure of performance, as this metric gives more weight to recall than precision (ie, it favours more conservative models that make FPs rather than FNs). The F2 score is defined as:


$$F_{2}=\frac{(1+2^{2})\times true\;positives}{(1+2^{2})\times true\;positives+false\;positives+2^{2}\times false\;negatives}$$

Precision is the proportion of true positives among all positive predictions (ie, positive predictive value) and recall is the proportion of correct positive predictions (ie, sensitivity). Model performance is presented in Results, while Model Bias Analysis focuses on model bias and data shift.

### Bias analysis and mitigation

We evaluate model bias through analysis of model performance on population subgroups, as well as with formalised measures of bias such as the Generalised Entropy Index (GEI).^41^ GEI is used to measure both group-based fairness (ie, how model performance differs between demographic groups) and individual fairness (ie, how the model treats patients who deserve similar outcomes). GEI uses the notion of benefit, which is defined as

*b_i_*=*M*(*x_i_*) - *y_i_*+1

where *y_i_* is the ground-truth outcome for a patient with features *x_i_* and *M*(*x_i_*) is the output of the predictive model. This definition gives patients with FN classifications the highest penalty, and patients with FP classifications the lowest. Benefit is calculated across the whole validation set (*b* = (*b*_1_*,b*_2_*,…,b_n_*), where *n* is the size of the validation set) to calculate the GEI as *I*^2^(*b*):


$$I^{\alpha }(b)=\frac{1}{n\alpha (\alpha -1)}\sum_{i=1}^{n}(\frac{b_{i}}{\mu }{)}^{\alpha }-1$$

where *µ* is the mean benefit across the whole validation set. This definition is then extended to measure between-group fairness where, for a given group of patients *g* ∈ *G* with *n_g_* patients, between-group fairness is defined as *I*^2^(*b*) where:


$$I_{\beta }^{\alpha }(b)=\sum_{g=1}^{\left|G\right|}\frac{n_{g}}{n\alpha (\alpha -1)}((\frac{\mu_{g}}{\mu }{)}^{\alpha }-1)$$

For the purposes of our experiments, we defined G based on the demographics of our validation cohort.

To investigate differences between patient populations across our four datasets, we extensively compare each feature’s distribution across all populations. Notably, we extend this to all available features rather than just patient demographics as is standard practice^42^; as explained in Supplementary Section 1, this analysis uncovered interesting (and previously unrecognised) differences in patient’s baseline creatinine values across the different study sites. To create an accurate picture of model performance on under-represented patients, we also evaluate model performance (using the same metrics as above) across patient subgroups to identify any groups where our techniques underperform (if, indeed, there are any such groups). For this, we use the patient’s reported sex, age (grouped into decades) and ethnicity (grouped using the UK’s Office for National Statistics (ONS) groupings^43^). However, in subgroups with extremely low patient numbers, these metrics can be misleading—a large enough sample size is needed in order to accurately estimate model performance. Therefore, we omit any subgroups with fewer than 10 samples.

We also experiment with using this subgroup aggregation as a bias mitigation technique, wherein we use broad ethnicity and treatment groups during model training and evaluation, rather than the exact ethnicity/treatment. To create the groups, we use the broad ethnicity categories defined by the ONS^43^ (eg, white, Asian), and group treatments by the number of regimens included singlet, doublet or triplet. The analysis of these methods, as well as the motivation behind their use, is in Subgroup Aggregation Strategies.

### Patient and public involvement

Through the development of our models, we conducted two focus groups with patients with cancer who have received chemotherapy. These were to understand the impact of blood monitoring on their lives and the acceptability of ML in practice. Patients were aware that data used in the development of models may not be representative, and therefore, supported the aims of these experiments.

## Results

There is a significant data shift between the three hospitals due to differing patient populations. Table 1 shows how patient age, sex, tumour type and number of creatinine/bilirubin grade changes differ between each of our four datasets. Supplementary Section 1 explores this data shift further, with detailed analyses of patient ethnicity, tumour incidences, age, and creatinine/bilirubin value distributions, as well as offering some hypotheses as to why these differences may exist. To allow for the analysis of how this data shift affects our models, we report results of evaluation on hold-out sets from hospitals 1A and 3, as well as the entire hospital 1B and hospital 2 datasets. Predicting Renal and Hepatic Dysfunction at Cycles 3-6 reports results of our risk stratification models trained to predict risk at cycles 3–6. Supplementary Section 2 provides an in-depth analysis of models trained on cycle 3 only, across different combinations of our training data, allowing for direct comparison with previous work.^31^

### Predicting renal and hepatic dysfunction at cycles 3–**6**

**Figure 1:**
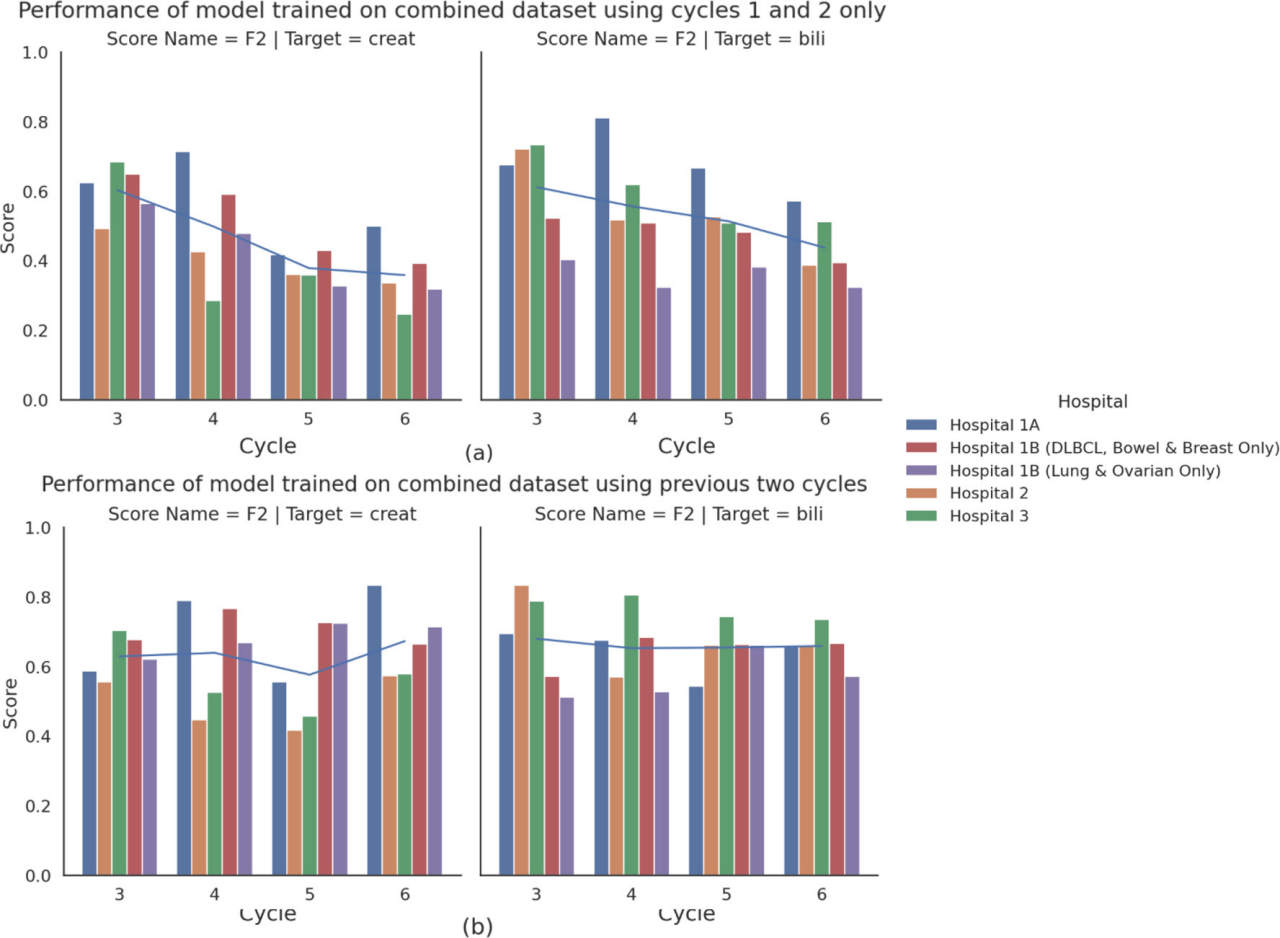
F2 metric across all predicted cycles of (A) models trained using data from cycles 1 and 2, and (B) models trained on cycles n−1, n−2. Hospital 1A, 2 and 3 data contain only tumour types seen by the models during training. Hospital 1B data have been stratified into two subgroups: records containing only cancer diagnoses during training (DLBCL, bowel and breast) and those that were not included during training (lung and ovarian). DLBCL, diffuse large B-cell lymphoma.

Figure 1a and online supplemental figure 4 present the performance of GBDTs predicting cycle n creatinine and bilirubin values based solely on demographics and cycle 1 and cycle 2 lab results (F2 creatinine: 0.4600, F2 bilirubin: 0.5294). As detailed in Method, models were tuned to have higher sensitivity than precision due to the greater potential harm of missed high-risk patients. This involved tuning decision thresholds for high-risk predictions, based on CTCAE guidance, to be more conservative; for creatinine the final grade boundaries are 1.4 × baseline or 1.4 × ULN, and for bilirubin the grade boundary used is 1.4 × ULN. Notably, model performance decreased with later cycles, likely due to the increase in time between the predicted cycle and the data available to the model. This effect is much more pronounced for creatinine than bilirubin.

On the other hand, figure 1b and online supplemental figure 5 show that models using data from the two cycles immediately preceding the prediction demonstrate strong performance across both creatinine and bilirubin (F2 creatinine: 0.6893, F2 bilirubin: 0.7773); although with the creatinine and bilirubin performance gap that is observed across all experiments. Unlike models using cycles 1 and 2 (figure 1a), performance remained stable throughout treatment, except for a slight dip in cycle 5 creatinine prediction. As these models were trained on a combined training set from hospitals 1 and 3, they performed consistently across all three validation sets despite their differing populations. Interestingly, they even slightly outperform those in online supplemental section 2 on cycle 3 prediction. We hypothesise that this stems from the larger training set that arises from treating each patient-cycle combination as a separate sample. This unified model likely learns features common across all cycles, leading to better generalisability. Figure 1b demonstrates our model’s robustness to temporal data shift; performance on Hospital 1B data is comparable to performance on all other data despite Hospital 1B data being collected significantly later than the other datasets. Additionally, the models successfully generalise to tumour types unseen during training; Figure 1b shows that performance on records with lung or ovarian cancers (which are unrepresented in the training data) is similar to that observed on DLBCL, bowel cancer and breast cancer data. The trade-off between precision and recall is seen in online supplemental table 2, where sensitivity (ie, recall) is higher than precision—this is also seen by the fact that the F2 scores are higher than the F1 scores across all models and targets.

## Model bias analysis

Despite strong performance (Results) and generalisability across hospitals, time and tumour types, some prediction failures remain. Analysing these failures is crucial both clinically (ensuring real-world effectiveness across diverse patient groups) and for understanding data shift. This section evaluates the perceived bias in the best-performing techniques presented in Predicting renal and hepatic dysfunction at cycles 3–6 (ie, prediction using the previous two cycles’ data) and examines how bias mitigation techniques may unintentionally affect model performance.

### Model fairness evaluation

**Figure 2:**
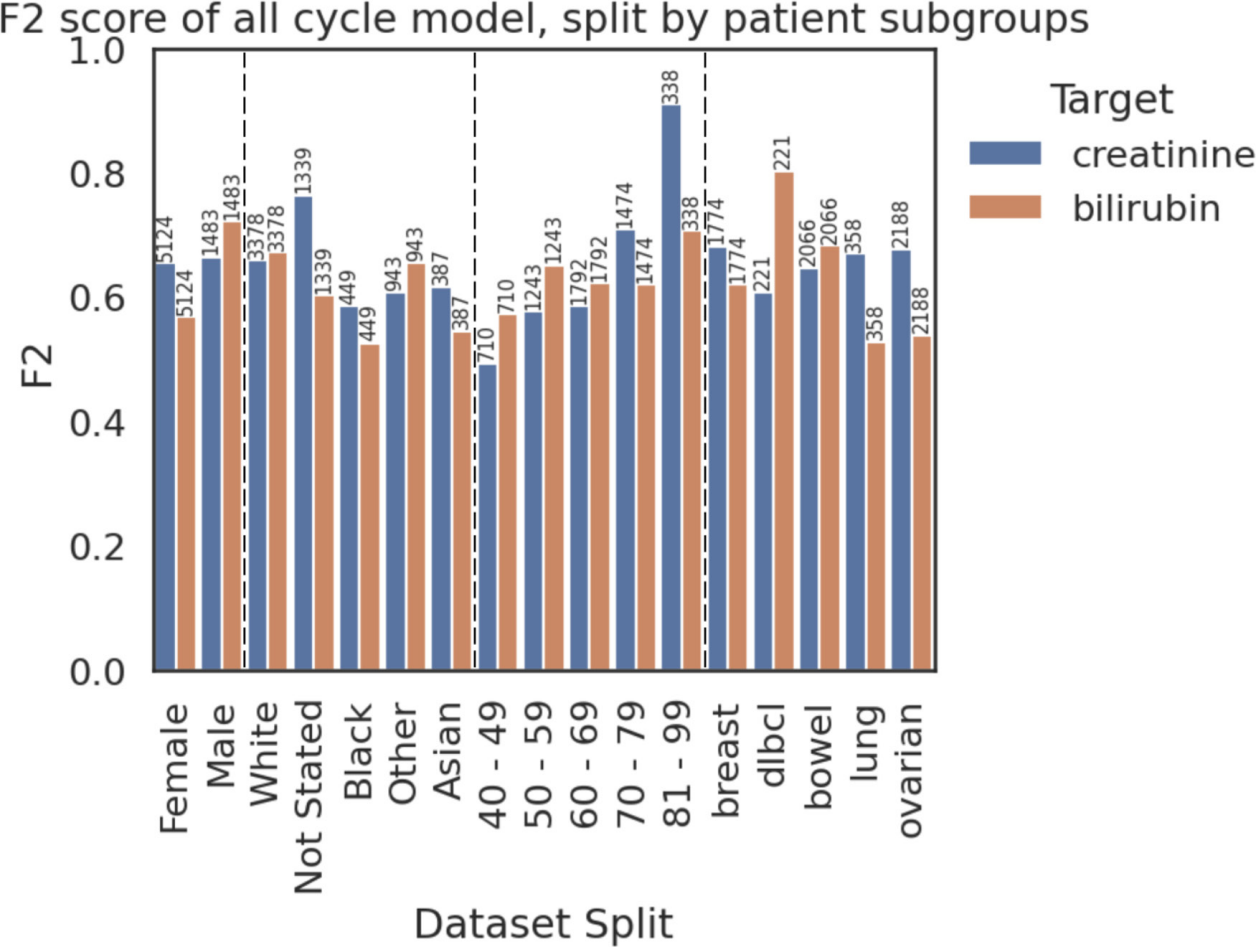
F2 score of each model on subgroups of the unseen combined test data. Dashed lines separate groups—from left to right they are: sex, ethnicity, age, cancer type. Bars are labelled with the number of test records in the subgroup, noting that there is a record for each individual cycle of treatment received (so a patient receiving five cycles of chemotherapy contributes five to the group count). Subgroups with fewer than 10 samples are not plotted.

Figure 2 highlights performance differences across patient subgroups; combined with hospital population knowledge, they can be used to draw numerous insights. For example, bilirubin prediction performs worse for women than men, and both creatinine and bilirubin models performed better on older patients. However, these trends are subtle and are likely due to limited training data size (especially in comparison to modern ML practice). Supporting this, both overall and subgroup-specific performance improved when training on hospital 1A and 3 data (as shown by Supplementary Figure 13); an encouraging sign for future studies which aim to collect more data for both model training and validation.

**Figure 3:**
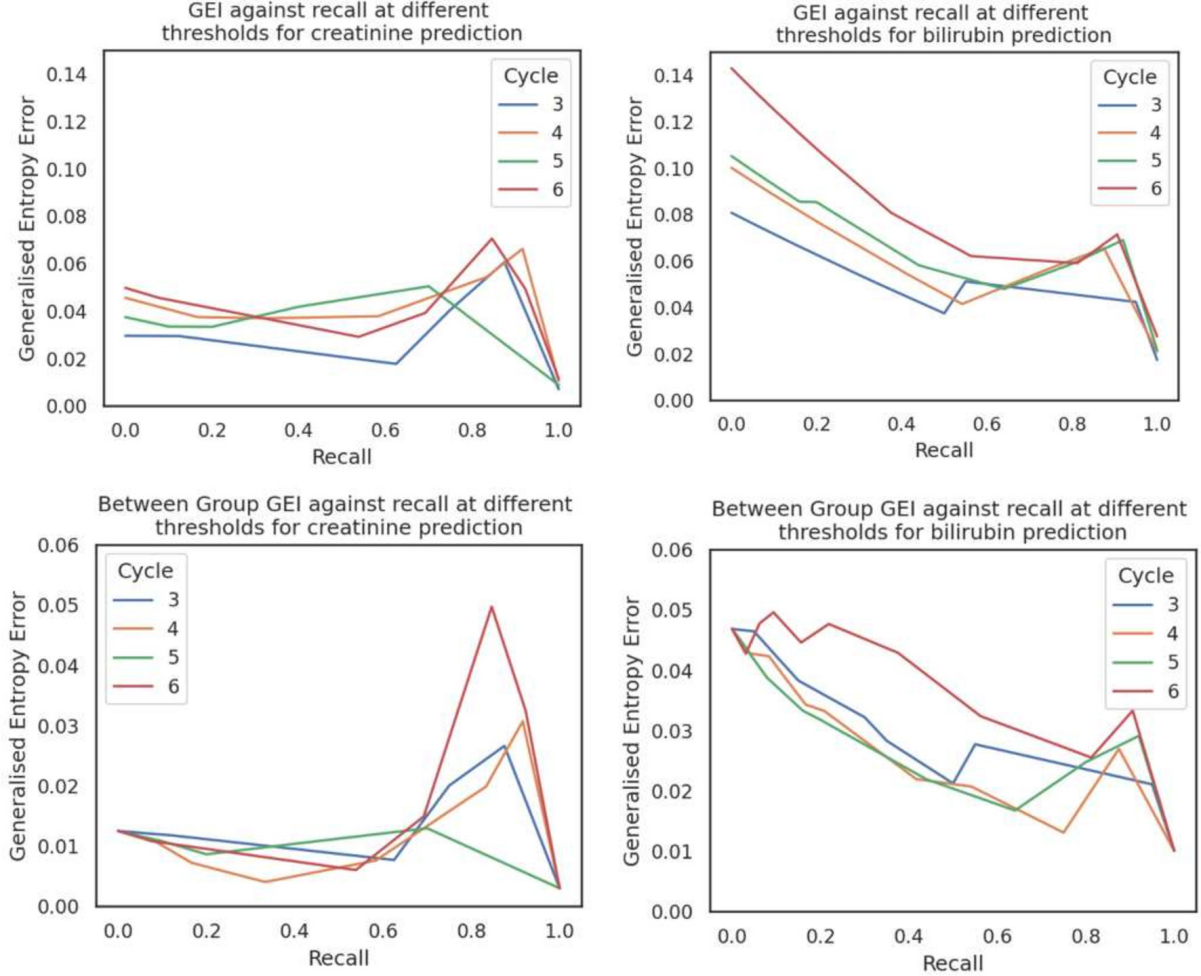
Generalised Entropy Index (GEI) (top) and between-group GEI (bottom) of each model across the whole unseen validation set, calculated at different thresholds. A perfect model would yield a single point at (0,1), the lower right-hand corner of the plot.

Figure 3 plots both *$I^{2}(b)\;and\;I_{\beta }^{2}(b)$* against sensitivity. All models showed low levels of unfairness, with $I^{2}(b)\leq 0.14$ and $I_{\beta }^{2}\leq 0.05$ across all cycles, with only small differences in estimated levels of fairness between cycles and only slight increases in later cycles. Conversely, the bilirubin model exhibits higher GEI than the creatinine model, aligning with the subgroup performance variations observed in figure 2.

### Subgroup aggregation strategies

**Figure 4:**
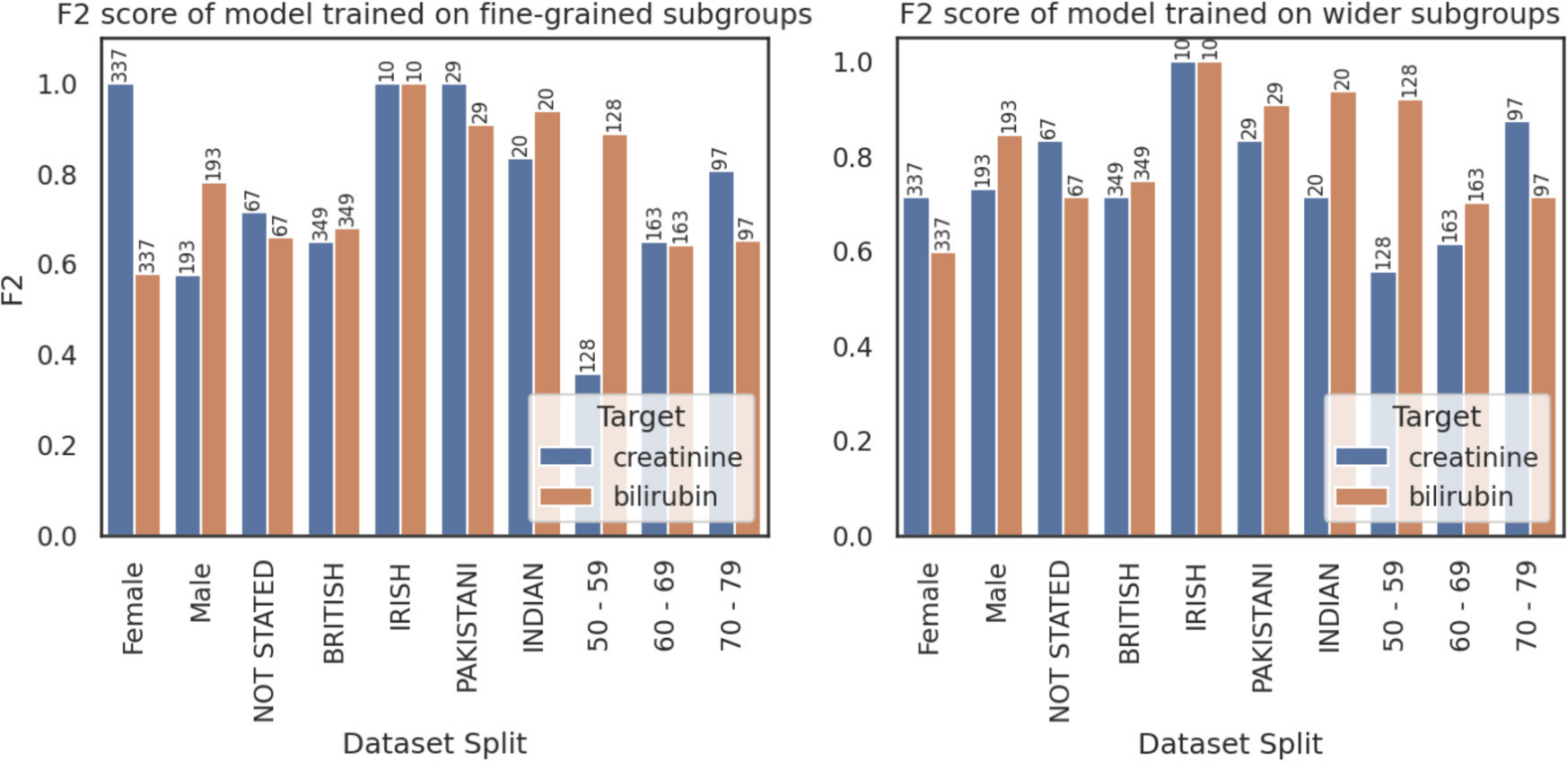
Comparison of model performance (on hospital 2 validation data) on patient subgroups of a model trained on fine-grained subgroups (left) and wider subgroups (right). Bars are labelled with the number of test records in the subgroup, noting that there is a record for each individual cycle of treatment received (so a patient receiving five cycles of chemotherapy contributes five to the group count). Subgroups with fewer than 10 samples are not included.

It is common to aggregate smaller subgroups into larger ones—for example, one may aggregate all white ethnic subgroups (English, Irish, British, etc) into a single white group.^43^ Similarly, chemotherapy regimens can be grouped by the type of treatment—that is, as doublet, triplet or quartet regimens. While this can both assist the analysis of results (by increasing the number of samples in each subgroup) and ease the burden of data collection, our experiments show it can alter healthcare model performance and biases. Figure 4, which compares models trained on fine-grained subgroups (eg, individual ethnicities) versus wider groups, highlights this phenomenon. While the overall performance of the two models was near-identical, subgroup performance varied significantly; as an example, the fine-grained model performed better on female patients and Indian/Pakistani patients. On the other hand, the broader group model performed better on older patients and male patients.

Although reasons for these discrepancies are not immediately clear and warrant future clinical work to investigate the interactions between patient demographics and outcomes, we can infer some possible relationships. For instance, the fine-grained model performed significantly better on female patients than its counterpart. This may stem from its high breast cancer performance—which could be due to more granular treatment information. Whereas the fine-grained model had information on the specific treatment regimen, the broad-subgroups model only knew regimen type (ie, doublet, triplet, quartet)—as all breast cancer treatments included in the study are doublet regimens, this could have led to significant information loss.

## Discussion

This work introduces models that predict creatinine and bilirubin changes across cycles 3–6, improving on and outperforming existing techniques^31^ focused solely on cycle 3. Notably, these experiments have shown that using only data from cycles 1–2 for later predictions is ineffective, suggesting deterioration can occur at any point during treatment and that early responses may not predict later ones. These long-term predictions are complex and hence require more data points. We observed that this effect is more pronounced for creatinine than bilirubin, possibly due to greater day-to-day variations in creatinine than bilirubin. However, we have shown that leveraging the previous two cycles accurately predicts current cycle renal/hepatic deterioration (F2 creatinine: 0.7423, F2 bilirubin: 0.6820). These high F2 scores, which are a result of high sensitivity values, indicate an extremely low number of FN results—crucial for ensuring patient safety were the model to be deployed in clinical practice. This can potentially improve the appropriateness of care when compared with current standards: predicted low-risk patients may not require precycle blood test results available immediately, and high-risk patients can thus undergo more intensive monitoring. Once validated and approved, our techniques would also significantly impact care in regions with limited phlebotomy services.^44 45^ Evaluation of our models on tumour types that were held out during model training highlights the generalisability of our technique and alleviates the need for costly data collection and model training across all possible cancer diagnoses.

This study underscores the significance of data shift for the broader healthcare ML community. We have shown that patient populations can vary significantly between hospitals and, in Model Bias Analysis, how this affects model training. We strongly believe that similar analyses should be standard practice in future medical ML projects, with projects moving beyond single-site studies and rigorously evaluating inherent data and model biases. Data shift is an unavoidable consequence of medical care as hospitals generally serve patients from their own catchment area, each with different demographic characteristics. As Model Bias Analysis has shown, data shift can significantly impact model performance on specific patient subgroups, and so addressing and understanding data shift is crucial before clinical deployment.

Furthermore, we have shown that common data aggregation strategies can disproportionately affect model bias—in both positive and negative ways. While they do simplify data collection and enable broader model deployment across non-standardised settings,^46^ our results show that care must be taken when deciding which features to aggregate, ensuring that they do not negatively affect the fairness or performance of the final model. Indeed, Model Bias Analysis proposes a standard set of analyses to detect and mitigate bias in medical ML studies, leading to successful final models:

1. A classical statistical analysis of the available dataset should be completed. Where possible, data should be collected from as many distinct sites as possible to ensure the diversity of the dataset.
2. Where studies are multisite, the data distributions for each individual site should be compared. If feasible, data from a whole site should be reserved for unseen validation.
3. A subset of data from each hospital used for training should be used as hold-out validation data. Performance metrics should be averaged across hospitals, to ensure reported metrics are not biased to a single site.
4. Model bias should be analysed through the use of both formalised fairness measures (eg, GEI), as well as the comparison of traditional performance metrics on at-risk and protected subgroups.
5. Where models are shown to work on some populations but not others (eg, due to data limitations), it may be appropriate to restrict deployment to suitable patient populations until future studies can be conducted (eg, our proposed technique is not suited to patients with abnormal baseline blood tests, as discussed in Model Bias Analysis).
6. Where data aggregation techniques are used, the effect on perceived model bias should be explored. Importantly, in clinical settings, any information loss that this may result in should be considered.

While many of these proposed analyses would be straightforward to implement, recent research showing the prevalence of biased ML models^18 47 48^ highlights their underutilisation. While reporting guidelines that are required by many journals prior to publication, such as Transparent Reporting of a multivariable prediction model for Individual Prognosis Or Diagnosis (TRIPOD)^42^ and its Artifical Intelligence (AI) variant, TRIPOD+AI,^49^ mandate thorough reporting of dataset statistics they do not go as far as to require formal model bias analysis and mitigation techniques. Importantly, our proposed analysis steps are specifically aimed at studies that evaluate ML models on data from multiple different clinical sites/populations with the aim of investigating model generalisability. As such, our proposed steps go above and beyond those required by TRIPOD, TRIPOD+AI and other similar reporting checklists. This paper has also conclusively shown that this type of analysis is necessary, and we hope future researchers find its recommendations useful.

## Conclusion

The main contributions of this paper are twofold. First, we have introduced refined risk stratification models for breast, bowel, lung, ovarian and DLBCL patients undergoing chemotherapy, accurately predicting renal and hepatic dysfunction across cycles 3–6. This advances beyond existing approaches focused solely on cycle 3, potentially enabling more appropriate care at later treatment stages. On cycle 3 prediction only, our proposed models outperform the current baseline techniques (supplementary Section 2. Our models have been shown to generalise to new cancer types that were not included in training data, potentially allowing for more widespread adoption. When compared with the current practice of requiring blood tests for all patients, at all cycles of treatment, the integration of our proposed model into clinical practice could both improve the appropriateness of care and reduce blood test-related delays to treatment. As chemotherapy services continue to be challenged with the growing number of patients with cancer and treatments available, the utilisation of ML is critical. Second, we have emphasised the prevalence of data shifts in healthcare and its impact on model fairness. We have proposed and demonstrated a gold-standard set of recommendations that should be followed when applying ML techniques to medical settings, showing that they can be successfully used to uncover and deal with biases present in the model.

We hope future work will extend the development of our chemotherapy risk stratification models to even more sites. This will address identified limitations and allow for model recalibration, aiming to reduce FPs while maintaining high sensitivity as achieved in this study. While our study has specifically evaluated model performance on unseen cancer types, data limitations meant this was only possible for lung and ovarian tumours. Future studies should extend this analysis to a wider range of cancers and treatment regimens to ensure wider clinical applicability. Furthermore, while our techniques are able to handle missing data, throughout our experiments, we have assumed that all blood tests are available—it would be prudent to evaluate model performance with increasing levels of missing data to better understand requirements for clinical deployment. We also hope that this work inspires future researchers to evaluate their techniques in similar ways, hopefully increasing the number of safe and effective ways that ML is applied (and subsequently deployed) to healthcare settings.

## Supplementary material

10.1136/bmjonc-2024-000430 online supplemental file 1

## Data Availability

Data are available on reasonable request.
