# Supplementary material for: From prediction to practice: mitigating bias and data shift in machine-learning models for chemotherapy-induced organ dysfunction across unseen cancers
Source: BMJ Oncol. 2024 Nov 2;3(1):e000430. doi: 10.1136/bmjonc-2024-000430 (PMC11557724; doi:10.1136/bmjonc-2024-000430)
Supplement: online supplemental file 1 [file bmjonc-2024-000430supp001.pdf]

1 The Presence of Data Shift

We collected data from three geographically distinct hospitals catering to diverse populations. This heterogeneity is reflected in Supplementary Figure 7, showing that Hospitals 2 and 3 had a slightly older population than Hospital 1, and that Hospital 2 had a wider range of ethnicities (though, notably, still mostly White) than the other two datasets. Supplementary Figure 7 also highlights how patient populations vary over time and across cancer diagnoses, with, for example, Hospital 1A patients being younger than Hospital 1B patients. Similarly, Supplementary Figure 8 shows that Hospital 3 had a much higher incidence of bowel cancer than the other sites - this in particular is important to note, as it could lead to models trained on this dataset to be biased towards bowel cancers. For cancers included in all data extractions (i.e. breast and bowel cancer), Hospital 1A and Hospital 1B had similar patient distributions, a result of data being extracted from the same hospital.

Beyond feature variable analysis, examining the impact of data shift on the target distribution is also crucial. Supplementary Figure 9 reveals that Hospital 3 had a significantly higher rate of renal deterioration than the other sites, primarily among breast and DLBCL patients (despite these patients making up a minority of patients for Hospital 3, as per Supplementary Figure 8). This potentially reflects Hospital 3 being a highly specialised cancer centre which is often referred more complex cases, or patients that may require more care; this is further supported by Supplementary Figure 10, which highlights that Hospital 3 breast and DLBCL patients generally had higher baseline creatinine values than the other patient groups, suggesting that these patients had a more complex condition than others. Although Supplementary Figure 7 shows that the patient population of Hospital 1 changed over time/with different cancer diagnoses, Supplementary Figures 9 and 10 reveals that renal and hepatic deterioration rates remained stable.

These variations in data distributions underscores the importance of thoroughly analysing training data, and necessitates evaluation of whether the data available is sufficient to conclusively say a proposed technique is suitable for deployment. Evidently, significant patient population variations exist both across hospitals and time, posing a potential for bias induction in models trained on this data. Differences in patient population is not the only issue, however - one must also consider differences in what data is collected and how it is measured. For instance, the way comorbidities are recorded in EP systems; Supplementary Figure 11 shows that comorbidity data for Hospital 3 was not available, and was also largely missing from Hospital 2. While incorporating these features is clinically relevant (these conditions may affect a patient's reaction to chemotherapy), the high proportion of missing values in the data could induce biases in the models. Although some techniques such as GBDTs are able to easily handle missing data, we argue that medical contexts demand additional consideration of what missing values signify. With comorbidities, for example, it could be assumed that patients that do not have a disease recorded (i.e. a missing diabetes value) do not have the disease - on the other hand, data collection in healthcare (particularly in acute care environments, or where links must be made between primary and secondary care) is not always reliable and it is just as likely that the data has not been recorded properly.

2 Predicting Renal and Hepatic Dysfunction at Cycle 3

While prior research has proposed models solely focused on predicting renal and hepatic dysfunction at cycle 3, our novel techniques offer a significant improvement by extending modelling to cycles 3-6. However, recognising the value of direct comparison, we additionally investigate the impact of data shift and bias on models specifically trained for cycle 3 prediction. Supplementary Table 1 and Supplementary Figure 13 both show the performance of the models trained for cycle 3 renal/hepatic dysfunction prediction.

As highlighted by Supplementary Figure 13, on the combined test set from all three hospitals, the cycle 3 models trained on Hospital 3, and Hospital 1 & 3 data, achieve a high F2 score of 0.8, with sensitivity being higher than precision - confirming that the models are able to accurately stratify risk for chemotherapy patients. Importantly, sensitivity is always high, meaning that the number of false negatives is low across all experiments - this is also highlighted by Supplementary Table 1. Table 1 in the main paper, as well as Supplementary Figure 9, also show that the proportion of patients who experience renal and/or hepatic deterioration is extremely low - with only around 10% of patients in these groups. Due to the relatively small size of the test sets, it is important to remember that this can result in a single mis-classification greatly affecting the evaluation metrics.

Supplementary Figure 13 also highlights the cycle 3 model performance on each of the separate test sets, allowing for the analysis of model generalisation to data from different hospitals. Supplementary Figure 13 is further evidence that there is some level of data shift occurring; models perform better on data from their own hospital than cycle 3 models trained on data from other hospitals. For instance, the top left plot of Supplementary Figure 13 shows that the cycle 3 model trained on Hospital 1 data is the best performing model on an unseen test set from Hospital 1, despite this model having significantly fewer training samples than the other two cycle 3 models.

Supplementary Figure 14 plots both $I^2(b)$ and $I^2_\beta(b)$ against sensitivity, calculated across multiple decision thresholds for the models trained for cycle 3 only. While all models are shown to be fair with $I^2(b) < 0.07$, cycle 3 models trained on data from both Hospitals 1 and 3 show greater fairness due to the wider range of patient populations this training set contains. This is particularly pronounced in between-group fairness, which is lower at almost all thresholds in models trained on data from both hospitals, providing further evidence for the data shift that occurs between hospitals and the need to train and evaluate on data from as many hospitals as possible.

3 Supplementary Tables

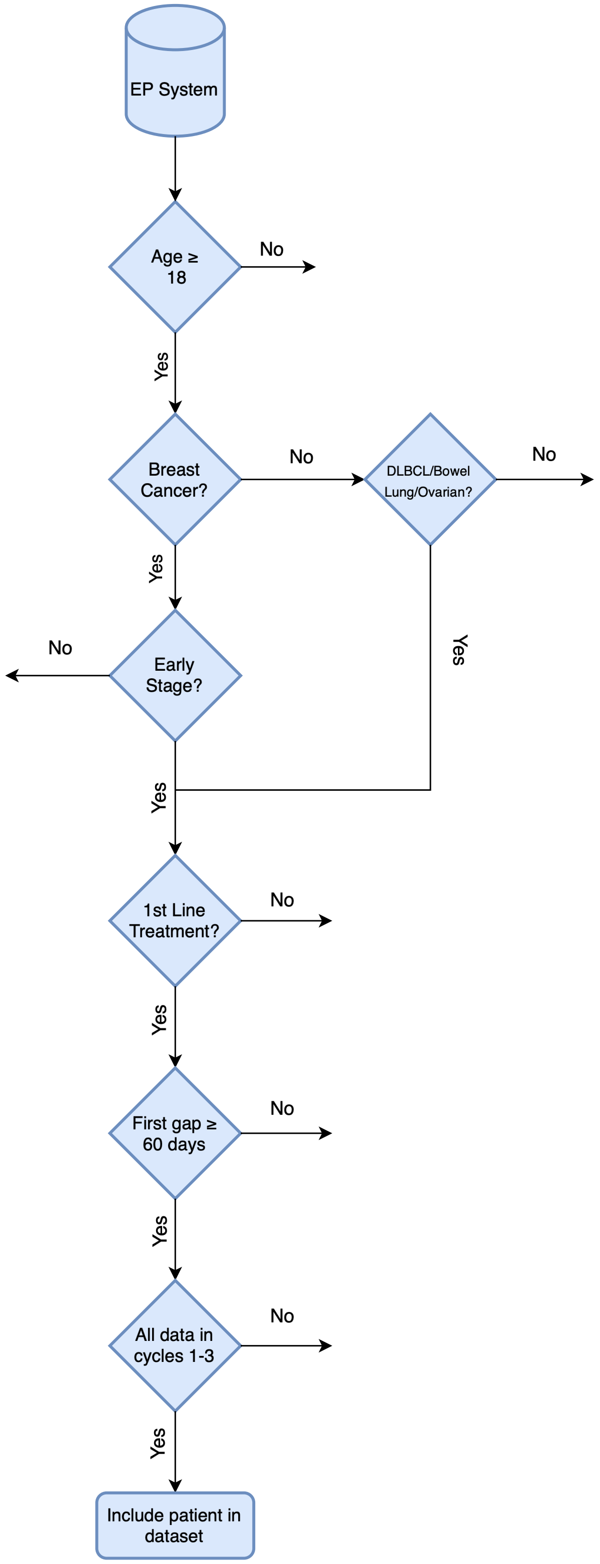

Supplementary Figure 1: Inclusion criteria applied to all three hospital's Electronic Prescribing (EP) systems for data extraction.

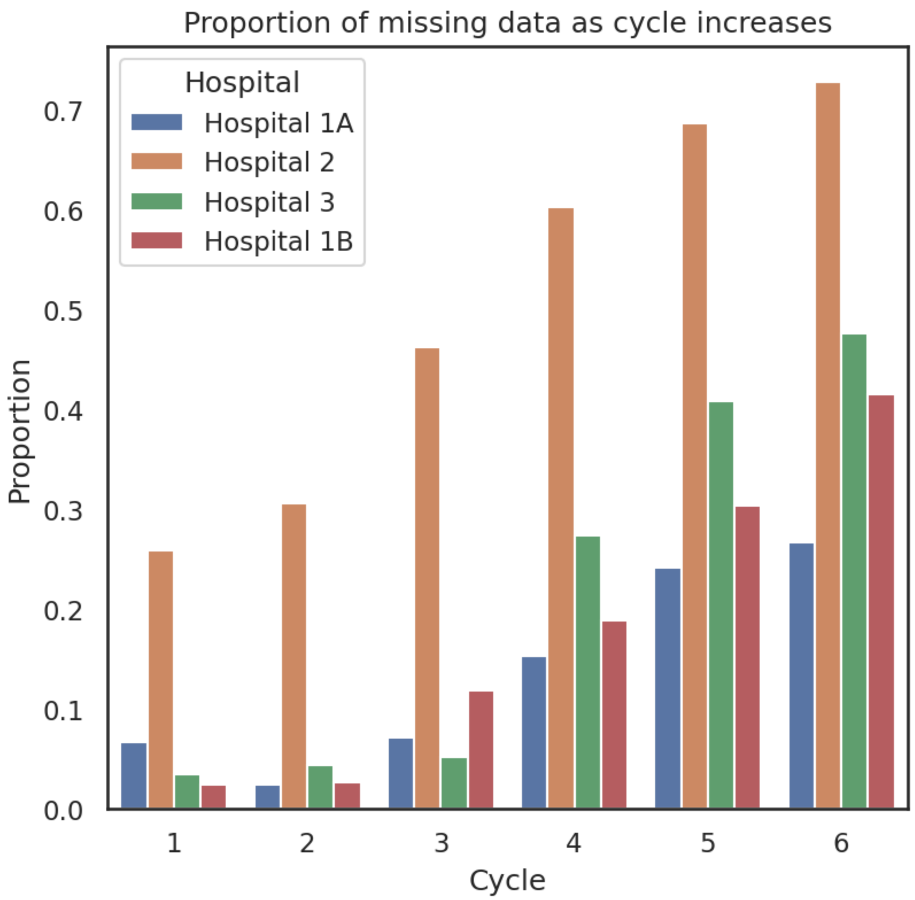

Supplementary Figure 2: Proportion of missing data per hospital as treatment progresses.

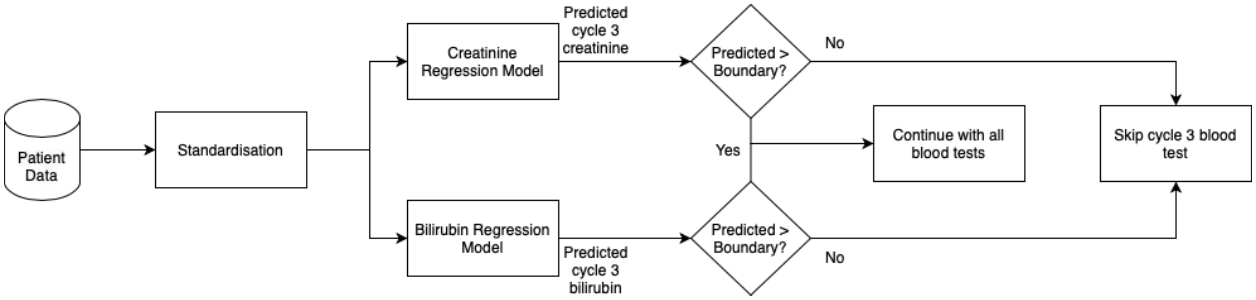

Supplementary Figure 3: Patient flow through our proposed system at cycle 3 of chemotherapy treatment.

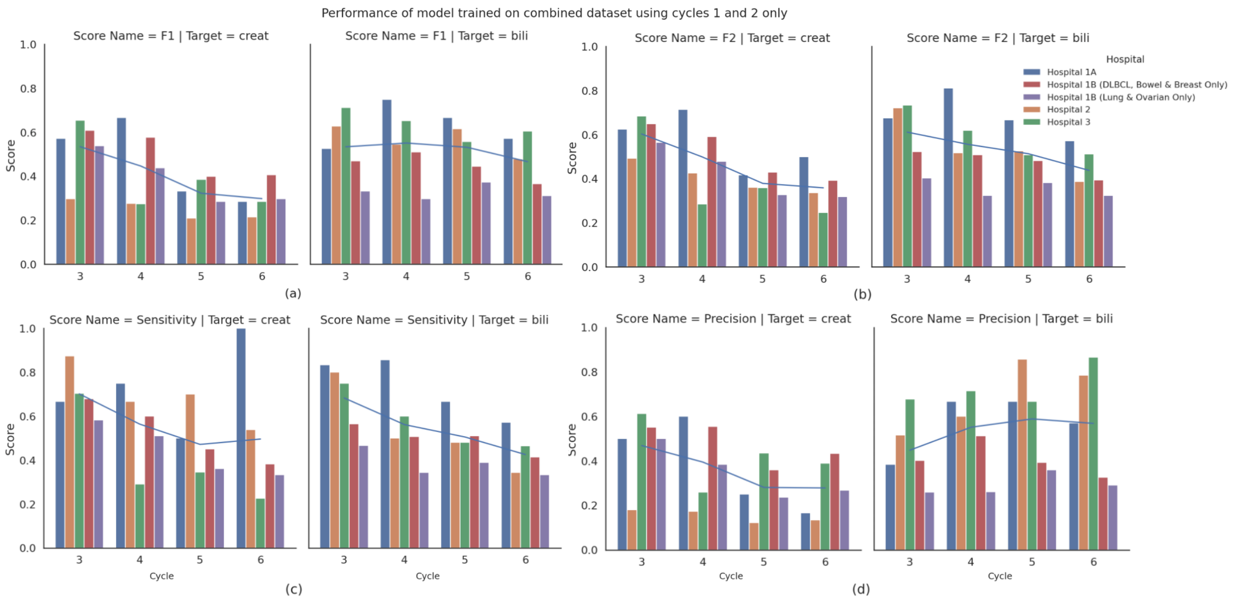

Supplementary Figure 4: (a) F1, (b) F2, (c) sensitivity, and (d) precision of GBDTs trained to predict creatinine (left) and bilirubin (right) levels at all cycles, using data from cycles 1 and 2 only.

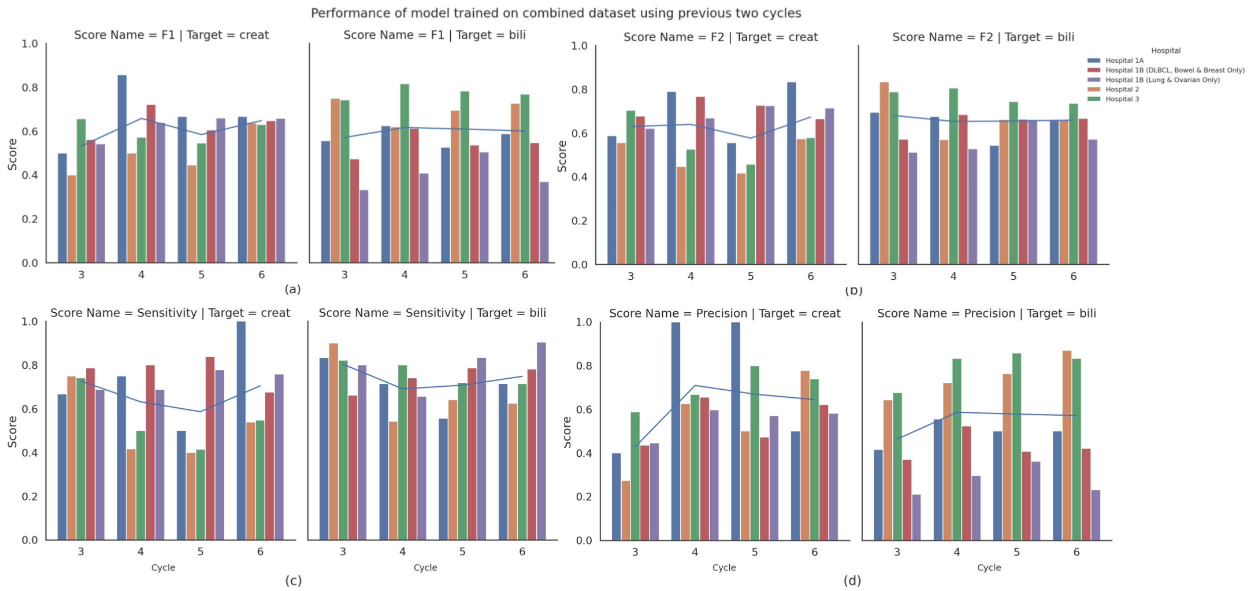

Supplementary Figure 5: (a) F1, (b) F2, (c) sensitivity, and (d) precision of GBDTs trained to predict creatinine (left) and bilirubin (right) levels of cycle n, using data from cycles $n-1$ and $n-2$.

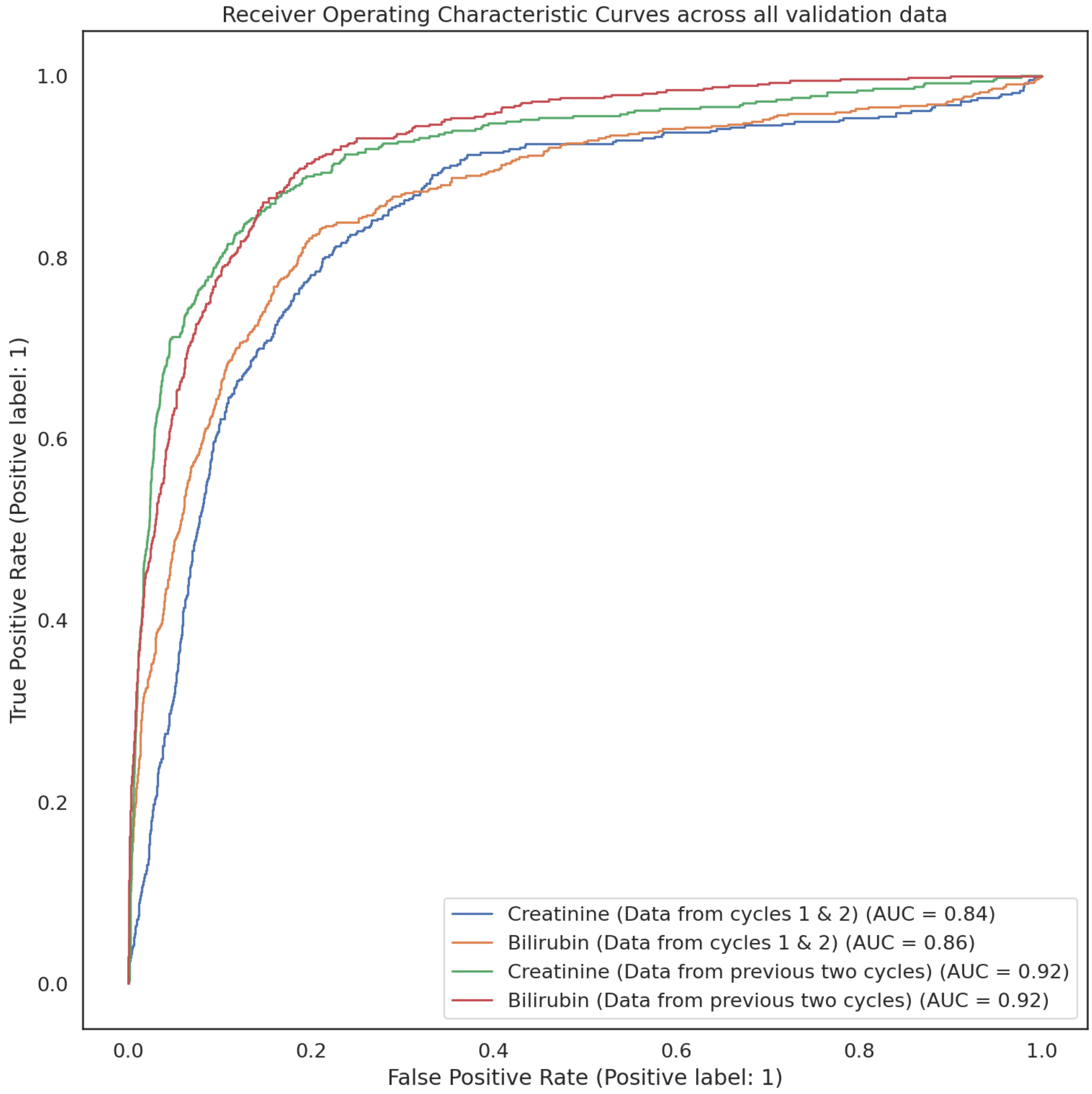

Supplementary Figure 6: Receiver Operating Characteristic (ROC) curves of both models using data from cycles 1 and 2, and models using data from cycles $n-1$, $n-2$ to predict cycle n. The ROC curve was computed using data from all validation sets.

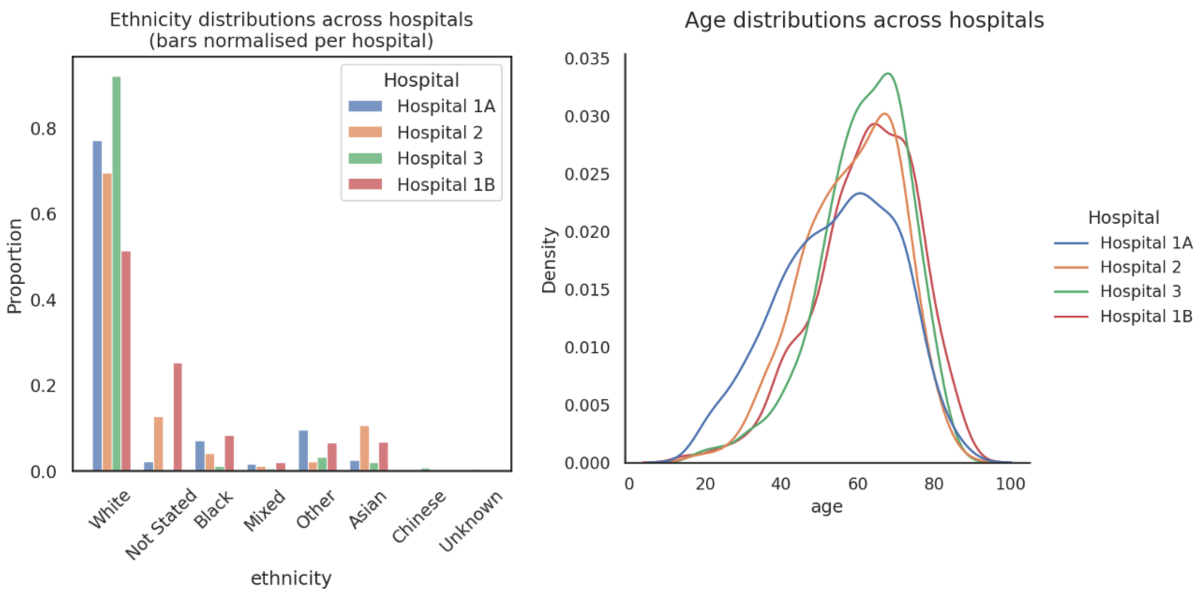

Supplementary Figure 7: Comparison of ethnicity (left) and age (right) distributions across all three hospitals.

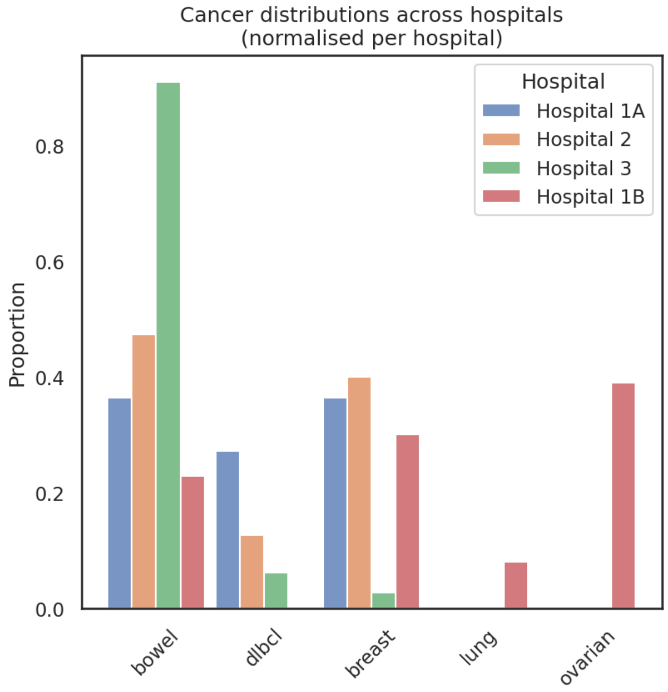

Supplementary Figure 8: Cancer distributions across each hospital.

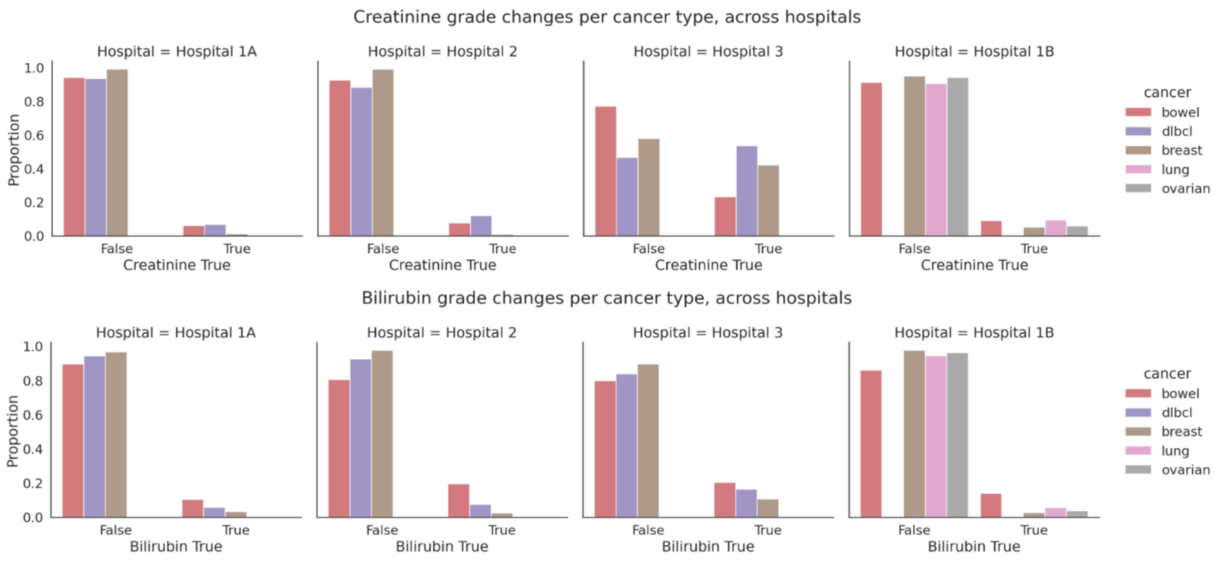

Supplementary Figure 9: Ground truth creatinine and bilirubin grade changes for each hospital, split by cancer type.

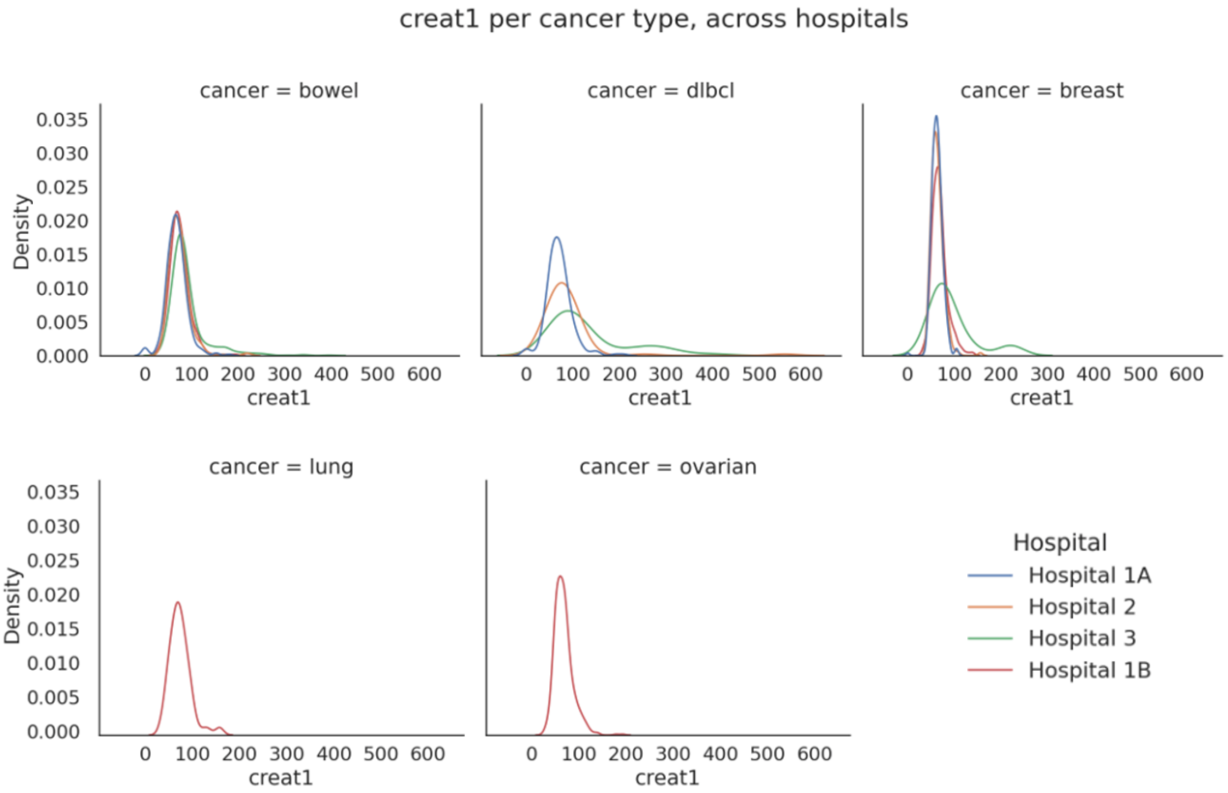

Supplementary Figure 10: Comparison of pre-cycle 1 creatinine value distributions for each cancer type, across each hospital in the study.

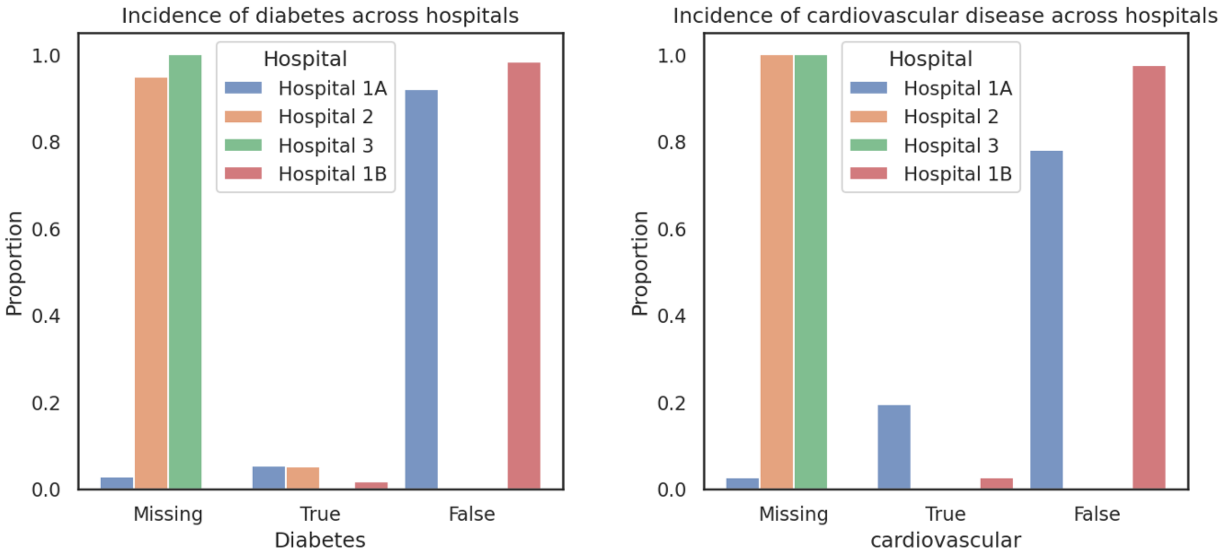

Supplementary Figure 11: Comparison of select comorbidity prevalence across the three hospitals in the study.

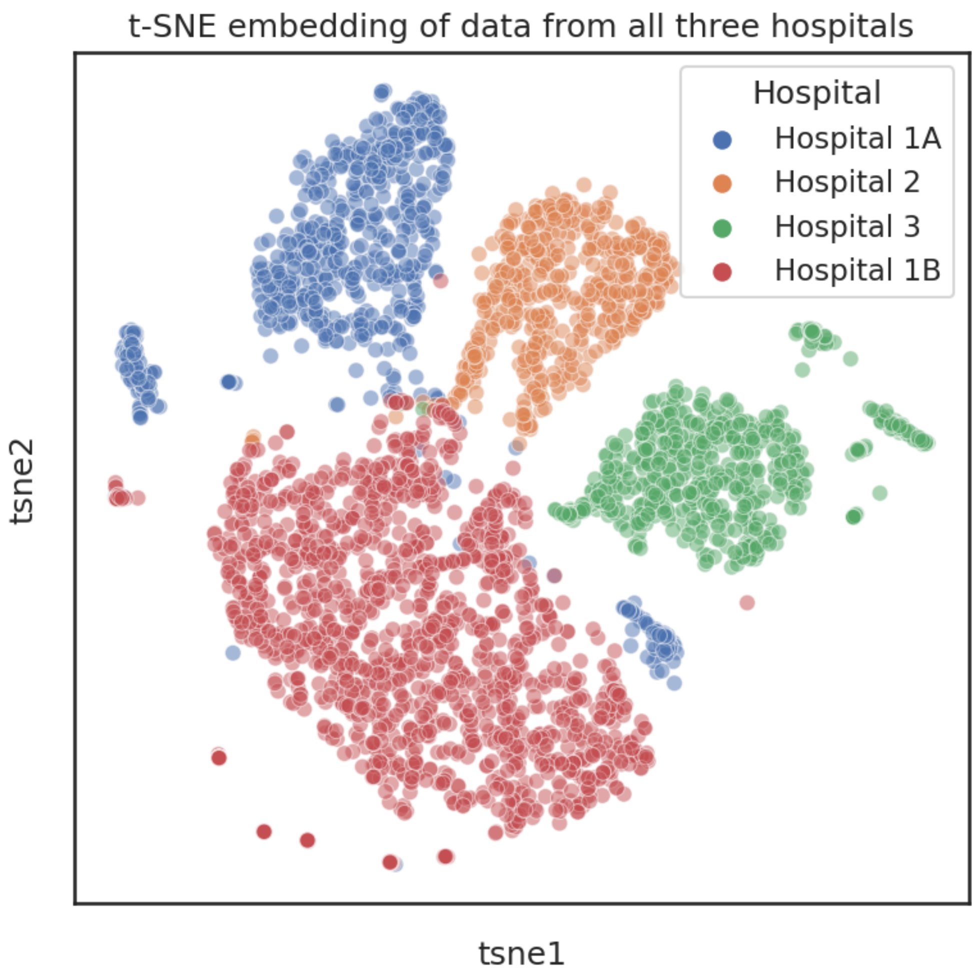

Supplementary Figure 12: 2D t-SNE embedding of a combined dataset containing all available data from the three hospitals included in the dataset.

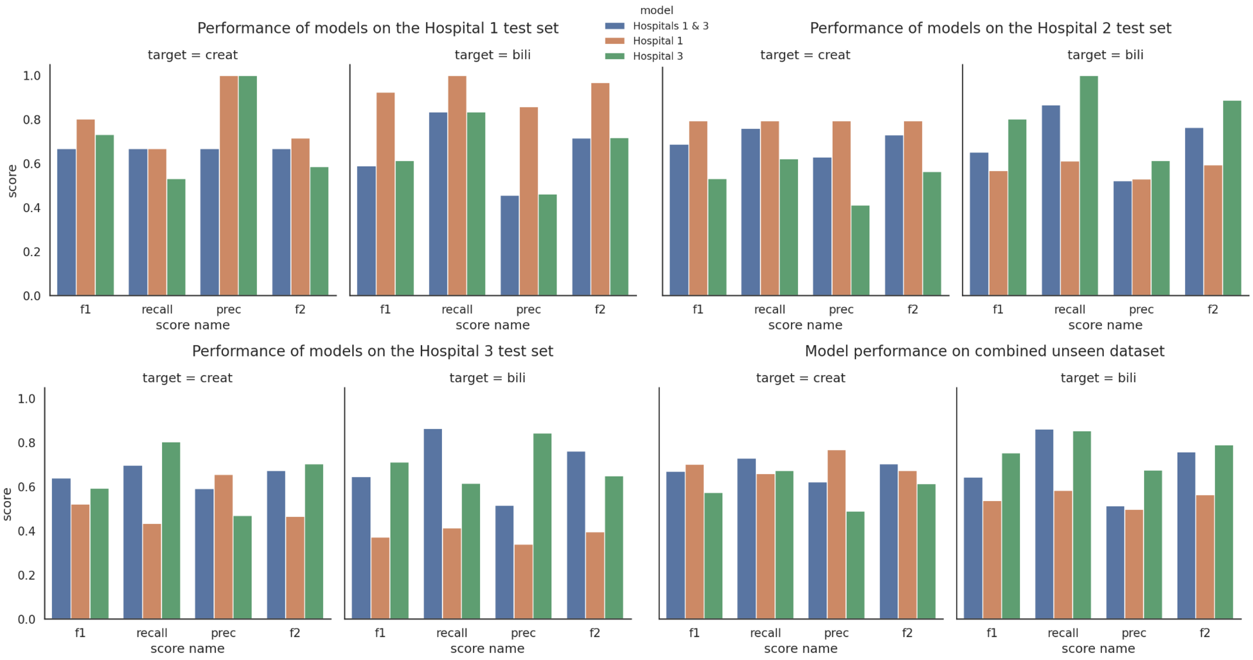

Supplementary Figure 13: F1, F2, sensitivity and precision of all three models trained for cycle 3 renal/hepatic dysfunction prediction across all three test sets, as well as a combined test set containing (unseen to all models) data from all three hospitals.

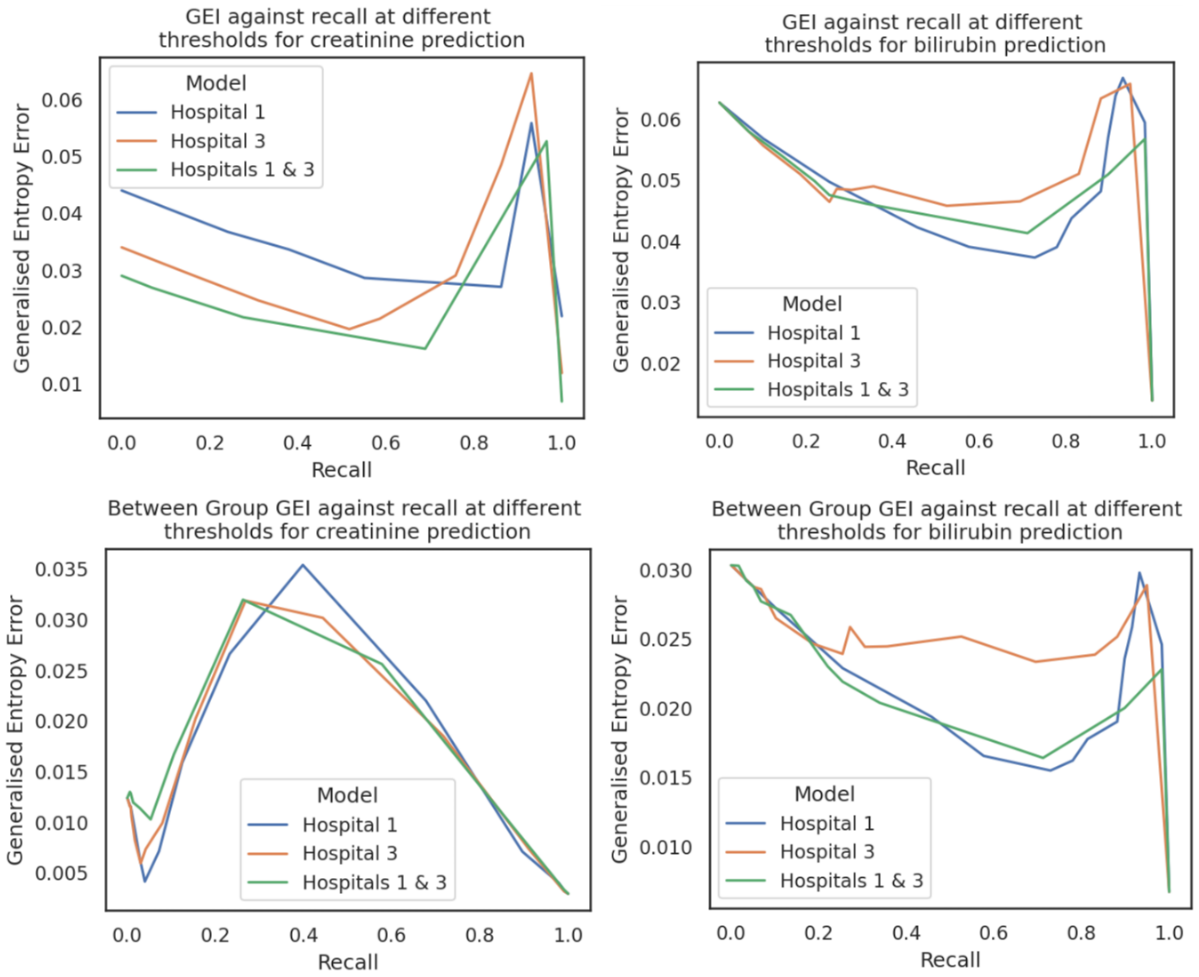

Supplementary Figure 14: Generalised Entropy Index (top) and Between-Group Generalised Entropy Index (bottom) of each cycle 3 dysfunction model across the whole unseen validation set, calculated at different thresholds. A perfect model would yield a single point at (0, 1), the lower right-hand corner of the plot.

Training Hospitals	Target	Test Set Hospital	Precision	Sensitivity	F1	F2	FNs
1 & 3	Creatinine	1	0.6666	0.6666	0.6666	0.6666	1
		2	0.6286	0.7586	0.6875	0.7284	6
		3	0.5909	0.6964	0.6393	0.6724	19
	Bilirubin	1	0.4545	0.8333	0.5822	0.7143	1
		2	0.5204	0.8644	0.6497	0.7635	14
		3	0.5135	0.8636	0.6441	0.7600	12
1	Creatinine	1	1.0	0.6666	0.8000	0.7142	1
		2	0.7931	0.7931	0.7931	0.7931	4
		3	0.6552	0.4318	0.5205	0.4634	100
	Bilirubin	1	0.8571	1.0	0.9231	0.9677	1
		2	0.5294	0.6102	0.5669	0.5921	7
		3	0.5294	0.4117	0.3709	0.4309	80
3	Creatinine	1	1.0	0.5304	0.7307	0.5854	0
		2	0.4093	0.6206	0.5313	0.5625	11
		3	0.4688	0.8036	0.5921	0.7032	11
	Bilirubin	1	0.4602	0.8333	0.6126	0.7170	1
		2	0.6122	1.0	0.8017	0.8876	0
		3	0.8438	0.6136	0.7105	0.6490	17

Supplementary Table 1: Full table of results for all model variations tested on cycle 3 grade change prediction.

Cycles Used	Target	Test Set Hospital	Precision	Specificity	Sensitivity	F1	F2
First Two	Creatinine	1A	0.3791	0.9558	0.7291	0.4642	0.5639
		2	0.1527	0.6900	0.6950	0.2495	0.4039
		3	0.4239	0.8567	0.3915	0.4000	0.3935
		1B (Bowel and Breast)	0.4746	0.9614	0.5281	0.4978	0.5150
		1B (Lung and Ovarian)	0.3470	0.9416	0.4472	0.3899	0.4221
	Bilirubin	1A	0.5723	0.9381	0.7321	0.6286	0.6811
		2	0.6897	0.9422	0.5309	0.5666	0.5379
		3	0.7312	0.9390	0.5735	0.6317	0.5935
		1B (Bowel and Breast)	0.4089	0.9338	0.4990	0.4474	0.4764
		1B (Lung and Ovarian)	0.2930	0.9542	0.3831	0.3288	0.3581
Last Two	Creatinine	1A	0.7250	0.9869	0.7291	0.6726	0.6916
		2	0.5438	0.9536	0.5262	0.4952	0.4981
		3	0.6985	0.9257	0.5507	0.6005	0.5666
		1B (Bowel and Breast)	0.5464	0.9541	0.7752	0.6334	0.7085
		1B (Lung and Ovarian)	0.5487	0.9585	0.7279	0.6242	0.6820
	Bilirubin	1A	0.4930	0.9204	0.7043	0.5737	0.6428
		2	0.7491	0.9522	0.6766	0.6979	0.6817
		3	0.8000	0.9438	0.7639	0.7775	0.7683
		1B (Bowel and Breast)	0.4299	0.9100	0.7423	0.5425	0.6461
		1B (Lung and Ovarian)	0.3619	0.8941	0.6431	0.4503	0.5423

Supplementary Table 2: Full table of results for models trained for the prediction of deterioration at cycles 3-6 (inclusive). Performance metrics are averaged across all 4 cycles.

Ethnicity \ Hospital	Hospital 1	Hospital 2	Hospital 3
White	482 (77%)	100 (69%)	1178 (92%)
Black	44 (6%)	6 (4%)	13 (1%)
Asian	16 (3%)	15 (11%)	35 (3%)
Mixed	10 (2%)	2 (1%)	7 ($<1\%$)
Not Stated	14 (2%)	18 (13%)	6 ($<1\%$)
Other	61 (10%)	3 (2%)	41 (3%)

Supplementary Table 3: Breakdown of ethnicities across all hospitals included in the study.

**Hyperparameter**	**Search Space**
Learning Rate	$[0.001, 0.5]$
Max Depth	$[3, 40]$
Min Child Weight	$\{0.01, 0.1, 0.5, 0.8, 1, 2, 4, 8, 9, 10\}$
Estimators	$[50, 2000]$
Subsample	$[0.5, 0.9]$
Alpha	$[0.0, 1.0]$
Lambda	$[0.0, 1.0]$
Colsample	$[0.0, 1.0]$

Supplementary Table 4: Table of hyperparameter search space.

Cycles Used	Target	Training Set	Learning Rate	Max Depth	Min Child Weight	Estimators	Subsample	Alpha	Lambda	Colsample
First Two	Creatinine (cycle 3 only)	Hospital 1 Only	0.0034	4	2	1016	0.2951	1.37e-05	4.18e-08	0.7316
		Hospital 3 Only	0.044	7	6	85	0.5567	7.54e-04	2.45e-06	0.4393
		Hospital 1 & 3	0.0033	30	8	977	0.5489	2.21e-05	0.0017	0.6124
	Bilirubin (cycle 3 only)	Hospital 1 Only	0.0041	16	9	858	0.2533	9.74e-07	1.61e-05	0.2160
		Hospital 3 Only	0.056	3	8	227	0.5454	8.74e-04	3.53e-06	0.4638
		Hospital 1 & 3	0.1309	4	7	88	0.6782	0.1158	5.94e-07	0.2498
	Creatinine (all cycles)	Hospital 1 & 3	0.0063	33	6	690	0.6618	6.65e-07	3.12e-05	0.9029
	Bilirubin (all cycles)	Hospital 1 & 3	0.0015	36	10	1964	0.5677	0.0041	0.6229	0.9627
Previous Two	Creatinine (all cycles)	Hospital 1 & 3	0.0285	4	10	653	0.4186	1.19e-05	0.1712	0.8263
	Bilirubin (all cycles)	Hospital 1 & 3	0.0021	22	9	1385	0.3612	3.89e-07	3.58e-08	0.4520

Supplementary Table 5: Table of hyperparameters for each trained model, found after performing a Tree Parzen Estimator search across the hyperparameter space.

List of Figures

1 Inclusion criteria applied to all three hospital's Electronic Prescribing (EP) systems for data extraction.

2 Proportion of missing data per hospital as treatment progresses. . . . .

3 Patient flow through our proposed system at cycle 3 of chemotherapy treatment. . . . .

4 (a) F1, (b) F2, (c) sensitivity, and (d) precision of GBDTs trained to predict creatinine (left) and bilirubin (right) levels at all cycles, using data from cycles 1 and 2 only. . . . .

5 (a) F1, (b) F2, (c) sensitivity, and (d) precision of GBDTs trained to predict creatinine (left) and bilirubin (right) levels of cycle n, using data from cycles $n-1$ and $n-2$. . . . .

6 Receiver Operating Characteristic (ROC) curves of both models using data from cycles 1 and 2, and models using data from cycles $n-1$, $n-2$ to predict cycle n. The ROC curve was computed using data from all validation sets. . . . .

7 Comparison of ethnicity (left) and age (right) distributions across all three hospitals. . . . .

8 Cancer distributions across each hospital. . . . .

9 Ground truth creatinine and bilirubin grade changes for each hospital, split by cancer type. . . . . . .

10 Comparison of pre-cycle 1 creatinine value distributions for each cancer type, across each hospital in the study. . . . .

11 Comparison of select comorbidity prevalence across the three hospitals in the study. . . . .

12 2D t-SNE embedding of a combined dataset containing all available data from the three hospitals included in the dataset. . . . .

13 F1, F2, sensitivity and precision of all three models trained for cycle 3 renal/hepatic dysfunction prediction across all three test sets, as well as a combined test set containing (unseen to all models) data from all three hospitals. . . . .

14 Generalised Entropy Index (top) and Between-Group Generalised Entropy Index (bottom) of each cycle 3 dysfunction model across the whole unseen validation set, calculated at different thresholds. A perfect model would yield a single point at (0, 1), the lower right-hand corner of the plot. . . . .
